# Supplementary material for: Restoring nuclear entry of Sirtuin 2 in oligodendrocyte progenitor cells promotes remyelination during ageing
Source: Nat Commun. 2022 Mar 9;13:1225. doi: 10.1038/s41467-022-28844-1 (PMC8907257; doi:10.1038/s41467-022-28844-1)
Supplement: Supplementary file 1 — Supplementary Information [file 41467_2022_28844_MOESM1_ESM.pdf]

Supplementary figures and figure legends

Supplementary Fig.1

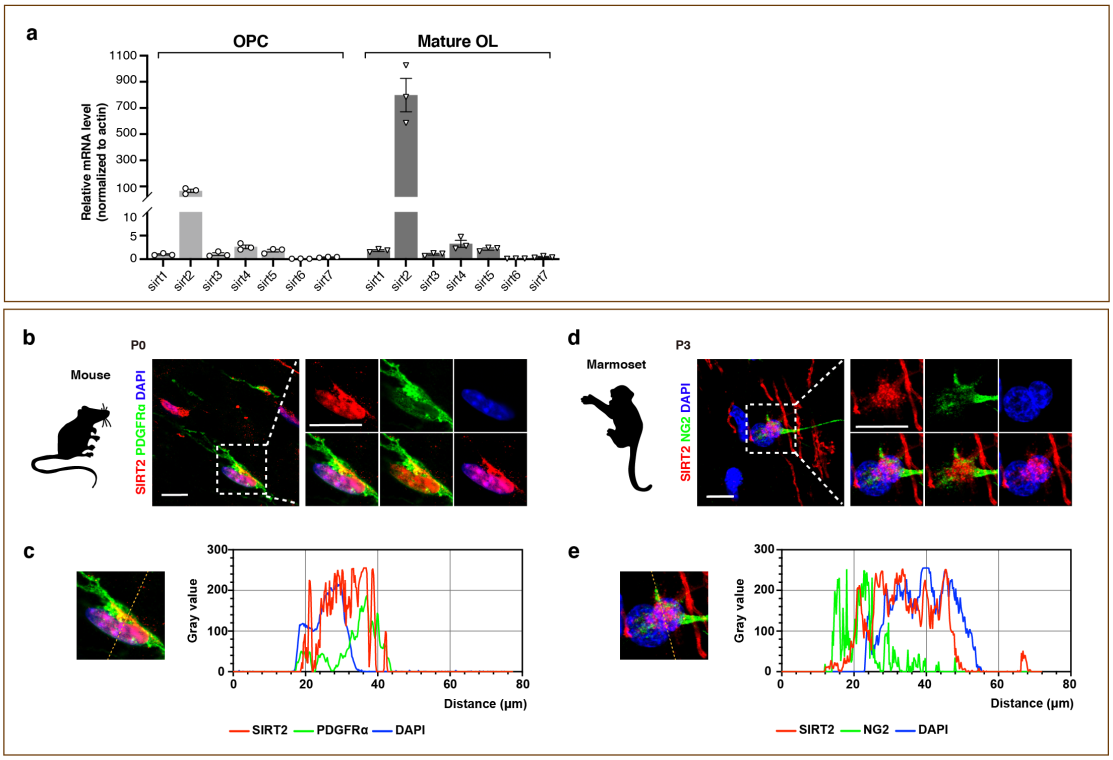

Supplementary Fig.1 | Nuclear localization of SIRT2 in OPCs in the brains of P0 mouse and P3 marmoset. a, qRT-PCR of 7 members of *sirtuins* in primary cultured mouse OPC and mouse mature oligodendrocyte (OL) from the cortex of P0 mouse (n=3). All groups were normalized to "OPC-*sirt1*" group. **b**, Immunofluorescence of SIRT2^{+} cells in P0 mouse. Scale bar, 20 µm **c**, Gray value of SIRT2, PDGFRα and DAPI. **d**, Immunofluorescence of SIRT2^{+} cells in P3 marmoset. Scale bar, 20 µm **e**, Gray value of SIRT2, NG2 and DAPI. All data are presented as mean ± SEM.

Supplementary Fig.2

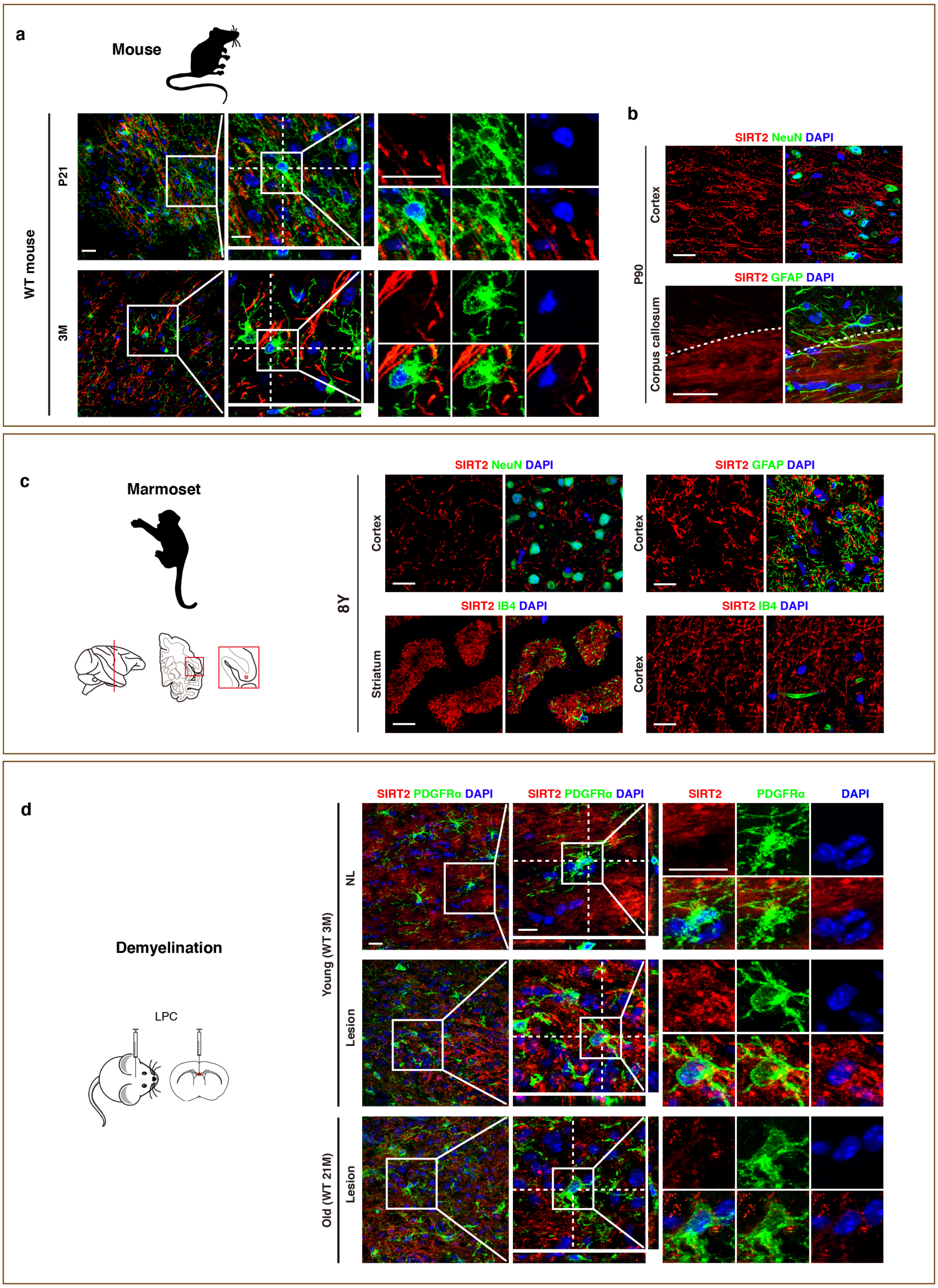

Supplementary Fig.2 | Characterization of SIRT2$^+$ cells in the brain during development, aging and after demyelination. a-b, images of SIRT2$^+$ cells in the cortex of mouse at different ages. Scale bar, 20 µm (far left panel images of **a**), 50 µm (rest panel images of **a**), 50 µm (**b**). **c**, images of SIRT2 in the brain of marmosets at age of 8 years. Scale bar, 20 µm. **d**, immunofluorescence of SIRT2$^+$ OPCs in corpus callosum of WT young or old mice (n=3). NL,

non-lesion, L, demyelination lesion induced by LPC at 5 dpl. Scale bar, 20 μm for the images on the far left and 50 μm for the rest.

Supplementary Fig.3

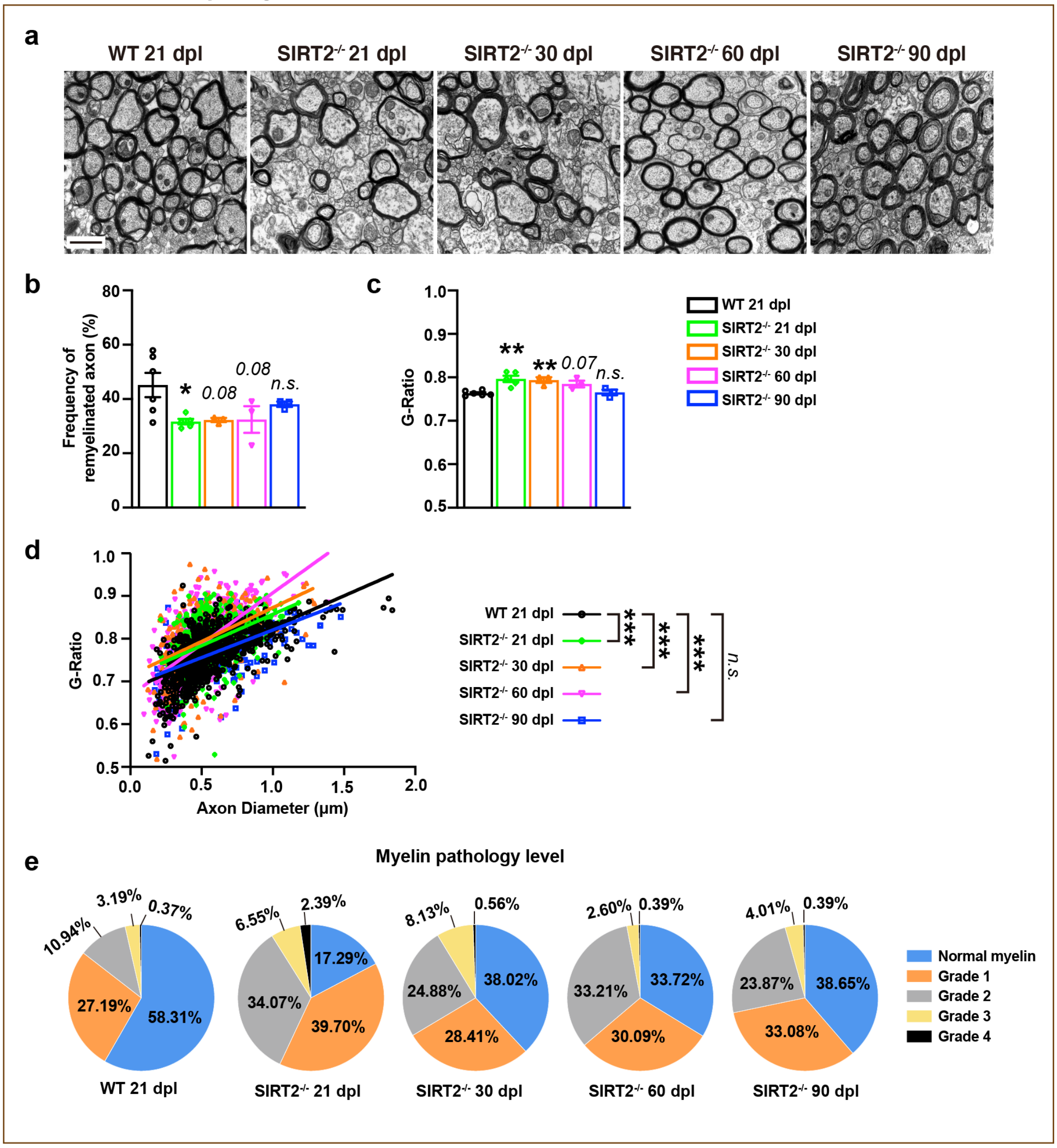

Supplementary Fig.3 | The impaired remyelination in SIRT2$^{-/-}$ mice is NOT a transient effect. a, TEM micrographs within the lesions. Scale bar, 1 μm. **b,** Quantification of the proportion of remyelinated axons. **c,** Quantification of G-Ratio. **d,** Quantification of individual G-Ratio distribution (linear regression). **e,** Quantification of myelin pathology level. n=6 for the WT 21 dpl group, n=5 for the SIRT2$^{-/-}$ 21 dpl group, n=3 for the SIRT2$^{-/-}$ 30 dpl group, n=3 for the SIRT2$^{-/-}$ 60 dpl group, n=3 for the SIRT2$^{-/-}$ 90 dpl group. All data are presented as mean ± SEM. *p<0.05, **p<0.01, ***p<0.001 by one-way ANOVA followed by Tukey's post hoc test (**b, c**). In all instances ***p<0.001. *n.s.* no significance. In (**b**), *p=0.03; In (**c**), **p=0.01 (WT 21 dpl vs. SIRT2$^{-/-}$ 21 dpl), **p=0.08 (WT 21 dpl vs. SIRT2$^{-/-}$ 30 dpl).

Supplementary Fig.4

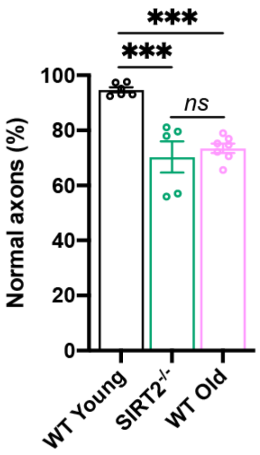

Supplementary Fig.4 I The percentage of normal-looking axons significantly decreased in SIRT2$^{-/-}$ and WT old mice. Quantification of the proportion of normal myelin in demyelinated lesion in corpus callosum of WT young, WT old, and SIRT2$^{-/-}$ mice at 21 dpl. n=5 in WT young mice group, n=5 in SIRT2$^{-/-}$ mice group, n=7 in WT old mice group. All data are presented as mean ± SEM. *p<0.05, **p<0.01, ***p<0.001 by one-way ANOVA followed by Tukey's post hoc test. In all instances ***p<0.001. *n.s.* no significance.

Supplementary Fig.5

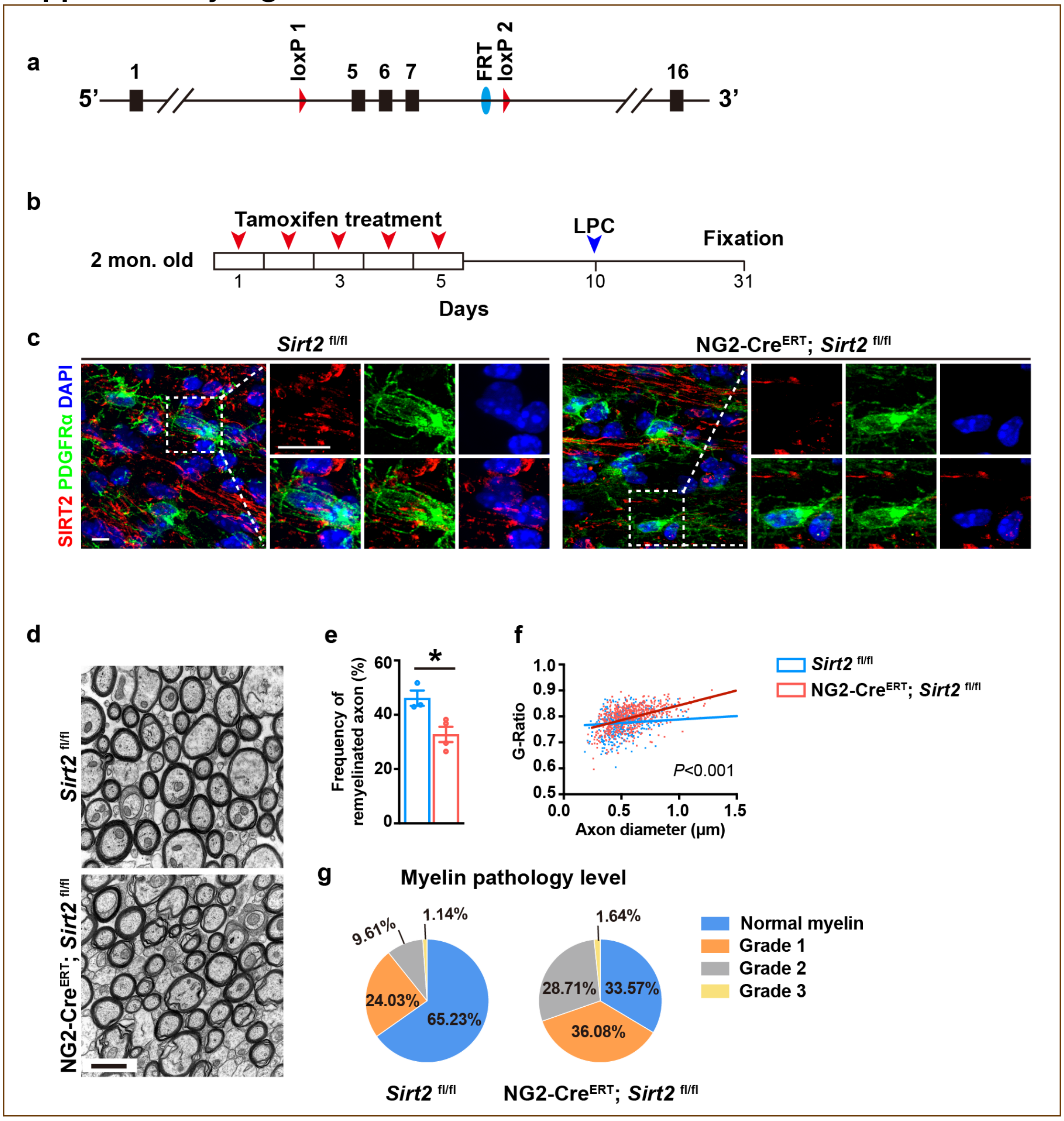

Supplementary Fig.5 | Conditional SIRT2 knockout specifically in OPCs of mouse verified that SIRT2 plays a critical role in remyelination. a, Schematic illustration of the conditional Sirt2 allele with loxP sequences (red) flanking exons 5–7 (black). The Flp recombinase recognition sequences (FRT site) are showed in blue. **b,** The flow chart of experimental design. Tamoxifen was given (100 mg/kg, intraperitoneal daily) for 5 days started at age of 2 months, and 4 days later, LPC was focally injected into the corpus callosum to induce demyelination. The mice were sacrificed at 21 days post LPC injection. **c,** Immunofluorescence of SIRT2 and PDGFRα+ OPCs within the demyelinated lesion in the corpus callosum of the *Sirt2*$^{flox/flox}$ mice and the NG2-Cre^{ERT}; *Sirt2*$^{flox/flox}$ mice at 21 dpl. Scale bar, 1 µm. **d,** TEM micrographs within the lesions at 21 dpl. Scale bar, 1 µm. **e,** Quantification of the proportion of remyelinated axons. **f,** Quantification of individual G-Ratio distribution (linear regression). **g,** Quantification of myelin pathology level. n=3 for the *Sirt2*$^{flox/flox}$ mice group, n=4 for the NG2-Cre^{ERT}; *Sirt2*$^{flox/flox}$ mice group. All data are presented as mean ± SEM. *p<0.05 by two-tailed t test (**e**). In all instances ***p<0.001. *n.s.* no significance. In (**e**), *p=0.02.

Supplementary Fig.6

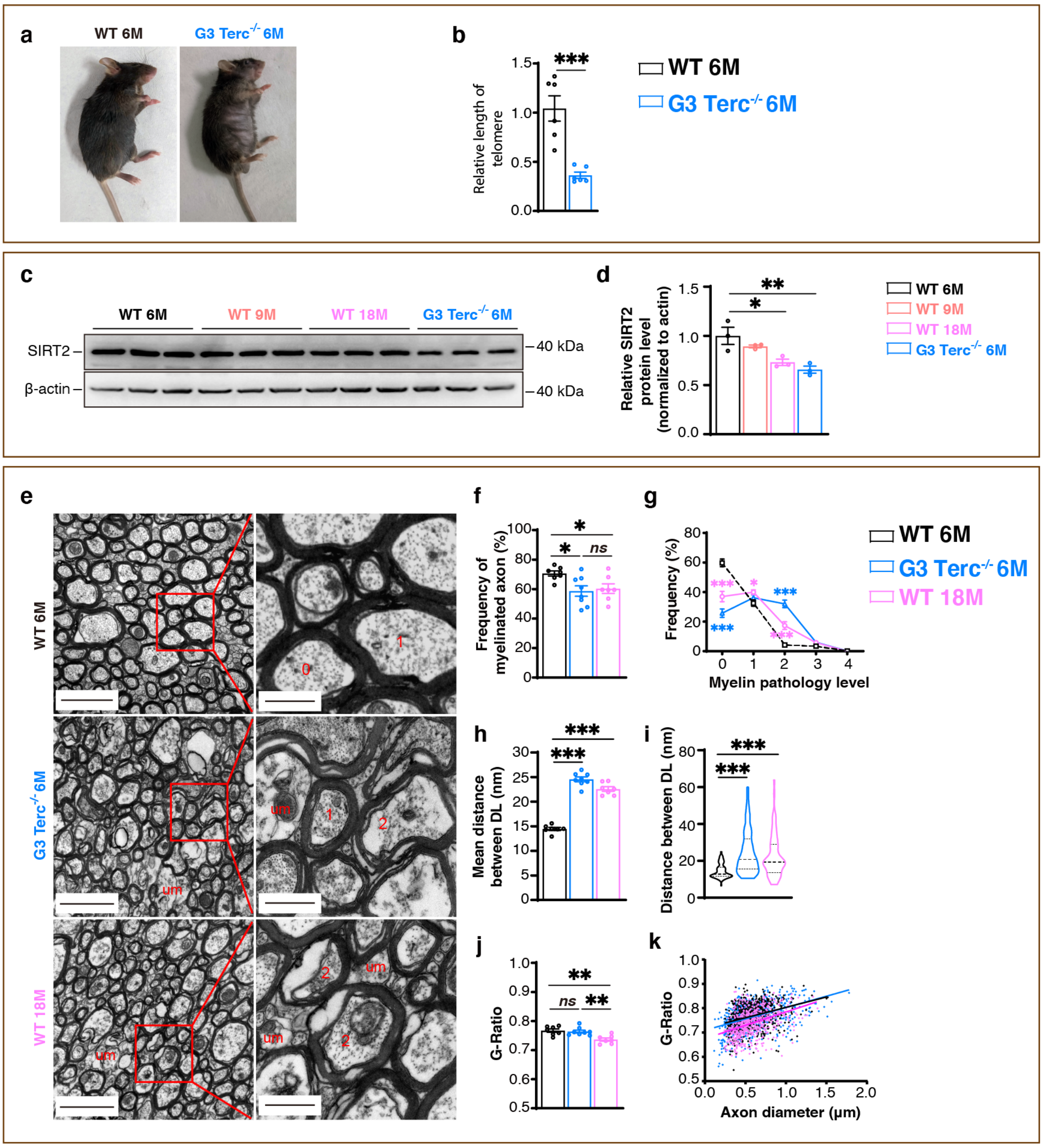

Supplementary Fig.6 | G3 $Terc^{-/-}$ mice exhibit premature myelin aging in the brain. a, Representative pictures of WT and G3 $Terc^{-/-}$ mice aged 6 months (6M). **b**, Relative telomere length of WT and G3 $Terc^{-/-}$ mice aged 5M (n=6). **c** and **d**, Relative SIRT2 protein level in brains of WT mice at different ages and G3 $Terc^{-/-}$ mice (n=3). **e**, Representative TEM micrographs of myelin in the corpus callosum of the brains of WT young (6M), G3 $Terc^{-/-}$ (6M) and WT old (18M) mice. um: unmyelinated axon. Scale bar: 2 μm for the left column images and 500 nm for the right column enlarged images. **f-k**, Quantification of the frequency of myelinated axons (**f**), myelin pathology level (**g**), distance between DL (**h** and **i**), G-Ratio average (**j**) and individual G-Ratio distribution (**k**, linear regression) in the corpus callosum of the brains of WT young (n=7), G3 $Terc^{-/-}$ (n=8) and WT old (n=7) mice. All data are presented as mean ± SEM. The center, upper and lower line represent the median, upper and lower quartiles, respectively (**i**). $^*p<0.05$, $^{**}p<0.01$, $^{***}p<0.001$ by two-tailed t test (**b**), one-way ANOVA followed by Tukey's post

hoc test (**d**, **f**, **h**-**j**) or two-way repeated ANOVA followed by Sidak's post hoc test (**g**). In all instances ***p<0.001. *n.s.* no significance. In (**d**), *p=0.02 (WT 6M vs. WT 18M), **p=0.006 (WT 6M vs. G3 Terc$^{-/-}$ 6M); in (**f**), *p=0.01 (WT 6M vs. G3 Terc$^{-/-}$ 6M), *p=0.02 (WT 6M vs. WT 18M); in (**g**), *p=0.03 (grade 1, WT 6M vs. WT 18M); in (**j**), **p=0.02 (WT 6M vs. WT 18M), **p=0.04 (G3 Terc$^{-/-}$ 6M vs. WT 18M).

Supplementary Fig.7

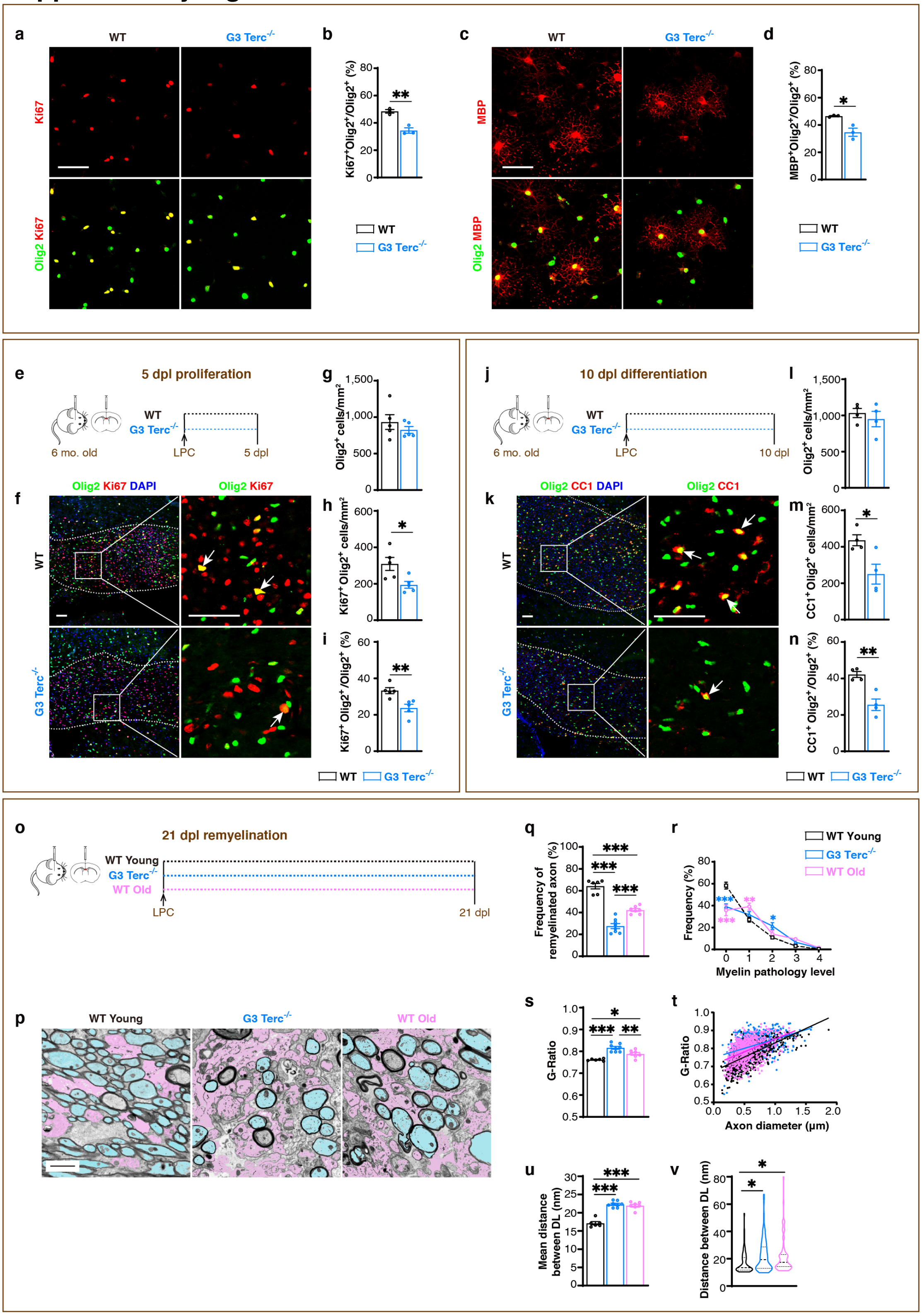

Supplementary Fig.7 | G3 Terc-/- mice exhibit declined capacity of OPCs on proliferation, differentiation and remyelination. a-d, Images and quantification of proliferating OPCs (Olig2+Ki67+, n=3) cultured for 36 hours and differentiated oligodendrocytes (Olig2+MBP+, n=3) cultured for 48 hours from P0 WT and G3 Terc-/- mice. Scale bar, 50 μm. **e**, Schematic diagram

of the experiment for testing OPC proliferation *in vivo*. **f**-**i**, Images and quantification of the densities of oligodendrocyte lineage cells (**g**, Olig2^{+}) and proliferating OPCs (**h**, arrows, Ki67^{+}Olig2^{+}) and the proportion of proliferating OPCs among all oligodendrocyte lineage cells (**i**) within the demyelination lesions (dotted line) at 5 dpl (n=5). Scale bar, 50 µm. **j**, Schematic diagram of the experiment for testing OPC differentiation *in vivo* at 10 dpl. **k**-**n**, Images and quantification of the densities of oligodendrocyte lineage cells (**l**, Olig2^{+}) and differentiated oligodendrocytes (**m**, CC1^{+}Olig2^{+}) and the proportion of differentiated oligodendrocytes among all oligodendrocyte lineage cells (**n**) within the demyelination lesions (dotted line) at 10 dpl (n=4). Scale bar, 50 µm. **o,** Experiment design for testing remyelination efficiency of WT young (6M), G3 Terc$^{-/-}$ (6M) and WT old (18M) mice *in vivo* at 21 dpl. **p**, TEM micrographs within the lesions at 21 dpl. Demyelinated axons are colored pink while remyelinated axons are painted blue. Scale bar, 1 µm. **q**-**v**, Quantification of remyelinated axons (**q**), myelin pathology level (**r**), G-Ratio average (**s**), individual G-Ratio distribution (**t**, linear regression) and distance between DL (**u** and **v**) within the lesions at 21 dpl (n=6 for the WT young group, n=8 for the G3 Terc$^{-/-}$ group, n=7 for the WT old group). All data are presented as mean ± SEM. The center, upper and lower line represent the median, upper and lower quartiles, respectively (**v**). *p<0.05, **p<0.01, ***p<0.001 by two-tailed t test (**b**, **d**, **g**-**i**, **l**-**n**), one-way ANOVA followed by Tukey's post hoc test (**q**, **s**, **u** and **v**) or two-way repeated ANOVA followed by Sidak's post hoc test (**r**). In all instances ***p<0.001. *n.s.* no significance. In (**b**), **p=0.0038; in (**d**), *p=0.017; in (**h**), *p=0.0221; in (**i**), **p=0.0064; in (**m**), *p=0.0251; in (**n**), **p=0.0035; in (**r**), **p=0.005 (grade 1, WT Young vs. WT Old), *p=0.01 (grade 2, WT Young vs. G3 Terc$^{-/-}$); in (**s**), *p=0.01 (WT Young vs. WT Old), **p=0.006 (G3 Terc$^{-/-}$ vs. WT Old); in (**v**), *p=0.02 (WT Young vs. G3 Terc$^{-/-}$ 6M), *p=0.04 (WT Young vs. WT Old).

Supplementary Fig.8

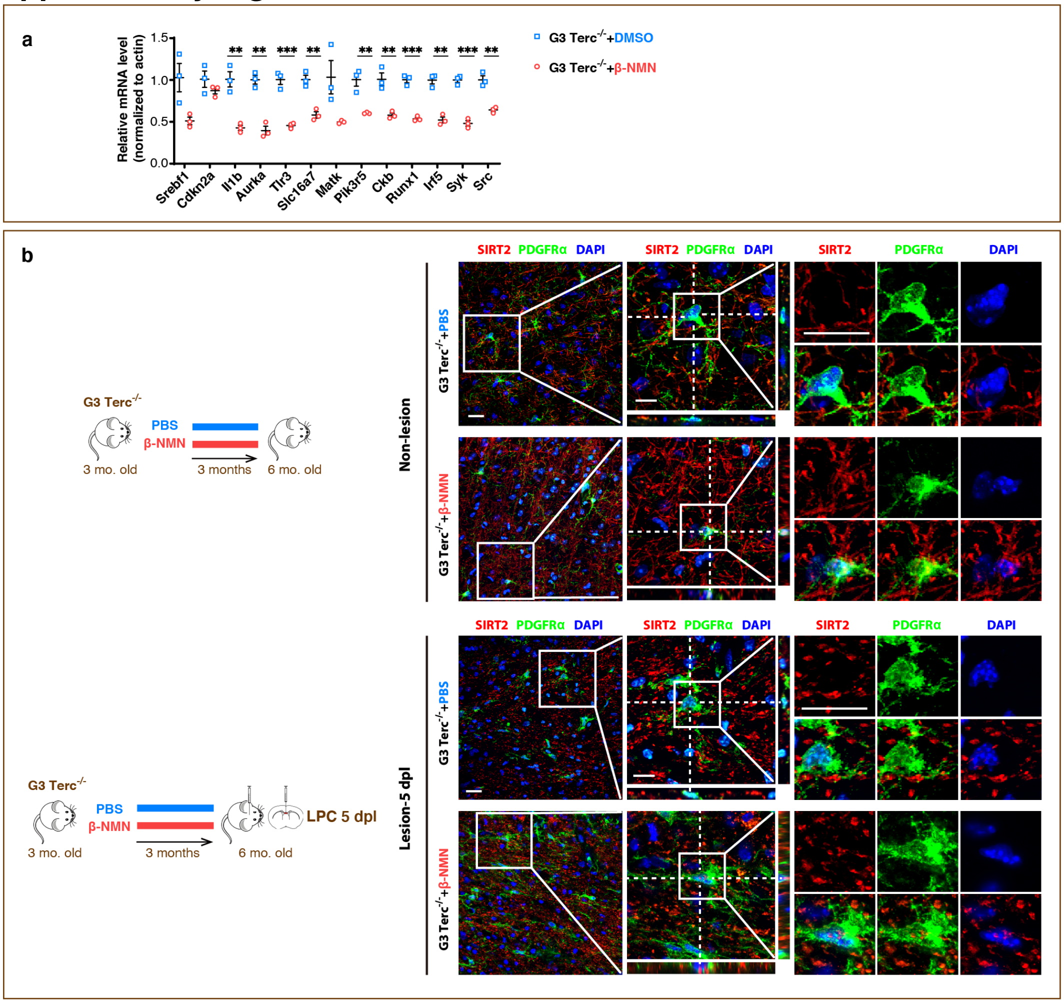

Supplementary Fig.8 | Elevating NAD^+ by β-NMN restores age-related decline of nuclear entry of SIRT2 in aged OPC within demyelination lesion. a, Relative mRNA level of cellular senescence related genes in primary cultured OPCs of G3 $Terc^{-/-}$ mice treated with or without β-NMN for 30 hours (n=3). **b**, Images of $SIRT2^+$ OPCs in corpus callosum of G3 $Terc^{-/-}$ mice with or without demyelination. Scale bar, 20 μm for the image on the far left and 10 μm for the rest. All data are presented as mean ± SEM. *p<0.05, **p<0.01, ***p<0.001 by two-tailed t test (**a**). In all instances ***p<0.001. *n.s.* no significance. In (**a**), **p=0.0038 (Il1b), **p=0.0012 (Aurka), **p=0.0028 (Slc16a7), **p=0.0065 (Pik3r5), **p=0.0061 (Ckb), **p=0.0012 (Irf5), **p=0.0023 (Src).

Supplementary Fig.9

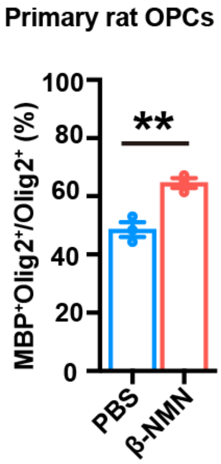

Supplementary Fig.9 I β-NMN promotes differentiation of primary cultured OPCs obtained from rat brain. Quantification of differentiated oligodendrocytes ($MBP^{+}Olig2^{+}$) cultured for 48 hours from P0 SD rats. All data are presented as mean ± SEM. *p<0.05, **p<0.01, ***p<0.001 by two-tailed t test. **p=0.006.

Supplementary Fig.10

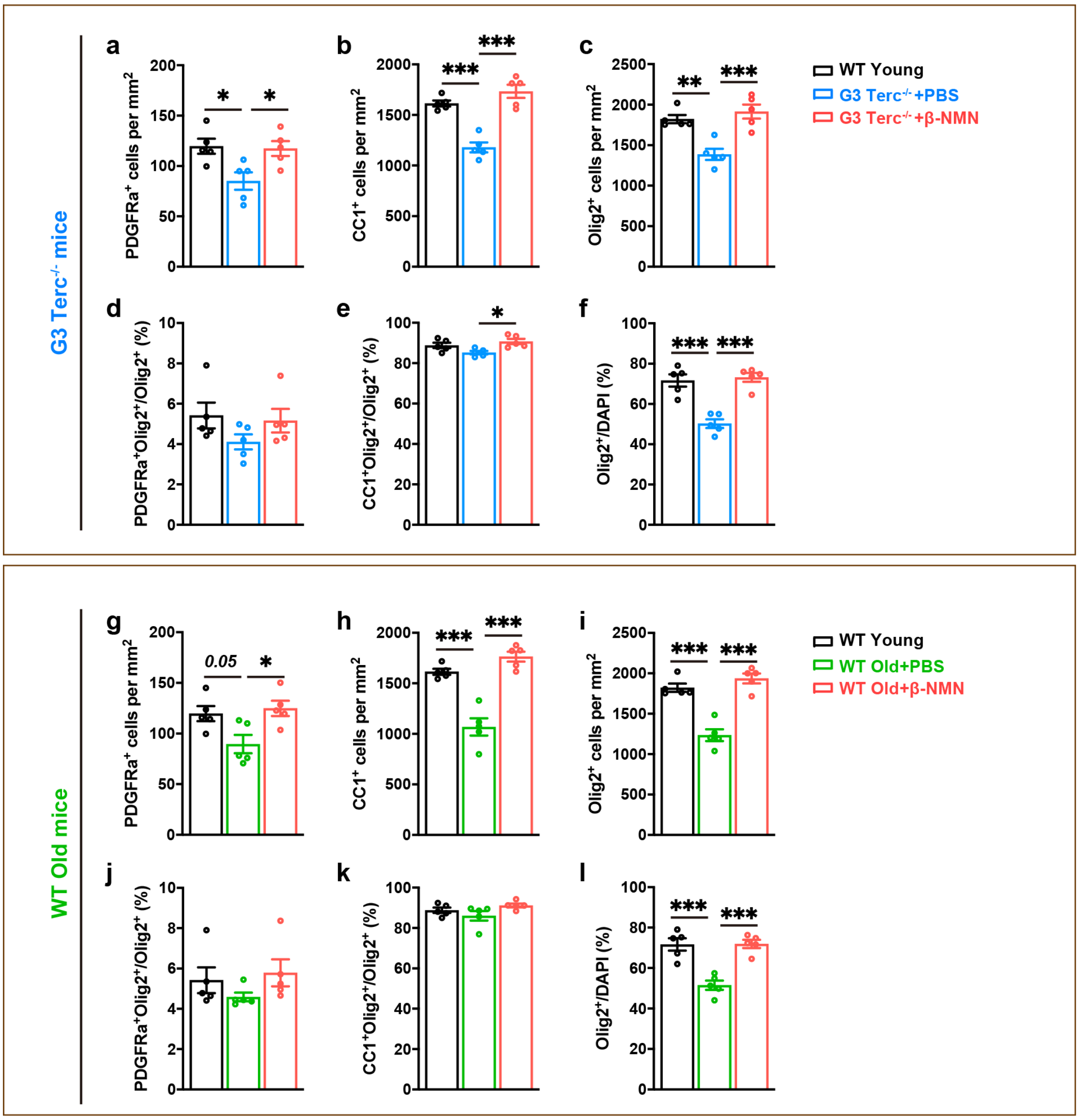

Supplementary Fig.10 I In non-lesion condition, β-NMN promotes the differentiation of OPCs and rejuvenates the aged OPCs in both G3 Terc$^{-/-}$ mice and WT old mice. a-f, Quantification of the density and the proportion of OPCs (a, d, PDGFRα^{+}Olig2^{+}), mature oligodendrocytes (b, e, CC1^{+}Olig2^{+}), and oligodendrocyte lineage cells (c, f, Olig2^{+}) in corpus callosum of the WT young mice and G3 Terc$^{-/-}$ mice. n=5 per group. **g-l,** Quantification of the density and the proportion of OPCs (g, j, PDGFRα^{+}Olig2^{+}), mature oligodendrocytes (h, k, CC1^{+}Olig2^{+}), and oligodendrocyte lineage cells (i, l, Olig2^{+}) in corpus callosum of the WT young and WT old mice. n=5 per group. All data are presented as mean ± SEM. *p<0.05, **p<0.01, ***p<0.001 by one-way ANOVA followed by Tukey's post hoc test. In all instances ***p<0.001. *n.s.* no significance. In (**a**), *p=0.02 (WT Young vs. G3 Terc$^{-/-}$+PBS), *p=0.03(G3 Terc$^{-/-}$+PBS vs. G3 Terc$^{-/-}$+β-NMN); in (**c**), **p=0.003 (WT Young vs. G3 Terc$^{-/-}$+PBS); in (**e**), *p=0.02 (G3 Terc$^{-/-}$+PBS vs. G3 Terc$^{-/-}$+β-NMN); in (**g**), *p=0.02 (WT Old+PBS vs. WT Old+β-NMN).

Supplementary Fig.11

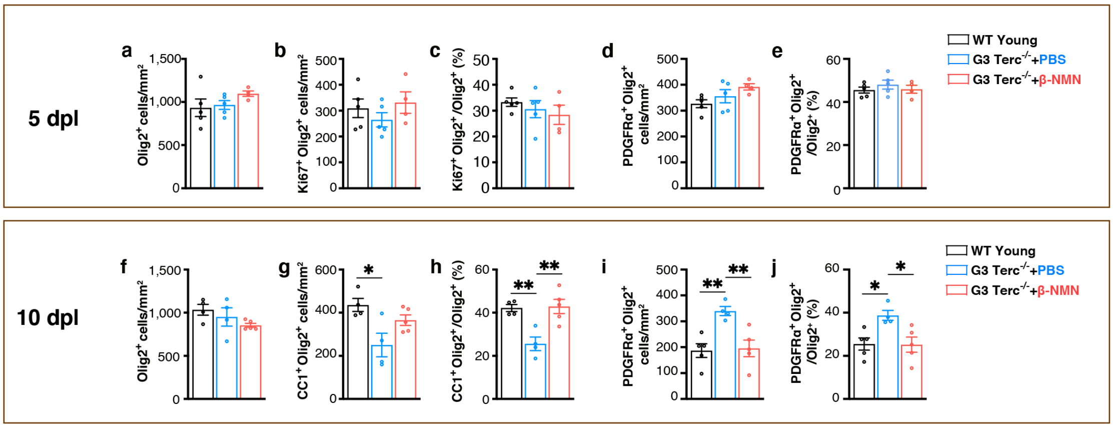

Supplementary Fig.11 | β-NMN promotes OPCs to differentiate into mature OLs in G3 Terc$^{-/-}$ mice. a-e, Quantification of oligodendrocyte lineage cells (**a**), proliferating OPCs (**b, c**) and total OPCs (**d, e**) within the demyelination lesions at 5 dpl (n=5 for the WT young group and the G3 Terc$^{-/-}$+PBS group, n=4 for the G3 Terc$^{-/-}$+β-NMN group). **f-j,** Quantification of oligodendrocyte lineage cells (**f**), differentiated oligodendrocytes (**g, h**) and total OPCs (**i, j**) within the demyelination lesion at 10 dpl (n=4 for the WT young group and the G3 Terc$^{-/-}$+PBS group, n=5 for the G3 Terc$^{-/-}$+β-NMN group). All data are presented as mean ± SEM. *p<0.05, **p<0.01, ***p<0.001 by one-way ANOVA followed by Tukey's post hoc test. In all instances ***p<0.001. *n.s.* no significance. In (**g**), *p=0.02 (WT Young vs. G3 Terc$^{-/-}$+PBS); in (**h**), **p=0.008 (WT Young vs. G3 Terc$^{-/-}$+PBS), **p=0.004 (G3 Terc$^{-/-}$+PBS vs. G3 Terc$^{-/-}$+β-NMN); in (**i**), **p=0.007 (WT Young vs. G3 Terc$^{-/-}$+PBS), **p=0.01 (G3 Terc$^{-/-}$+PBS vs. G3 Terc$^{-/-}$+β-NMN); in (**j**), *p=0.03 (WT Young vs. G3 Terc$^{-/-}$+PBS), *p=0.03 (G3 Terc$^{-/-}$+PBS vs. G3 Terc$^{-/-}$+β-NMN).

Supplementary Fig.12

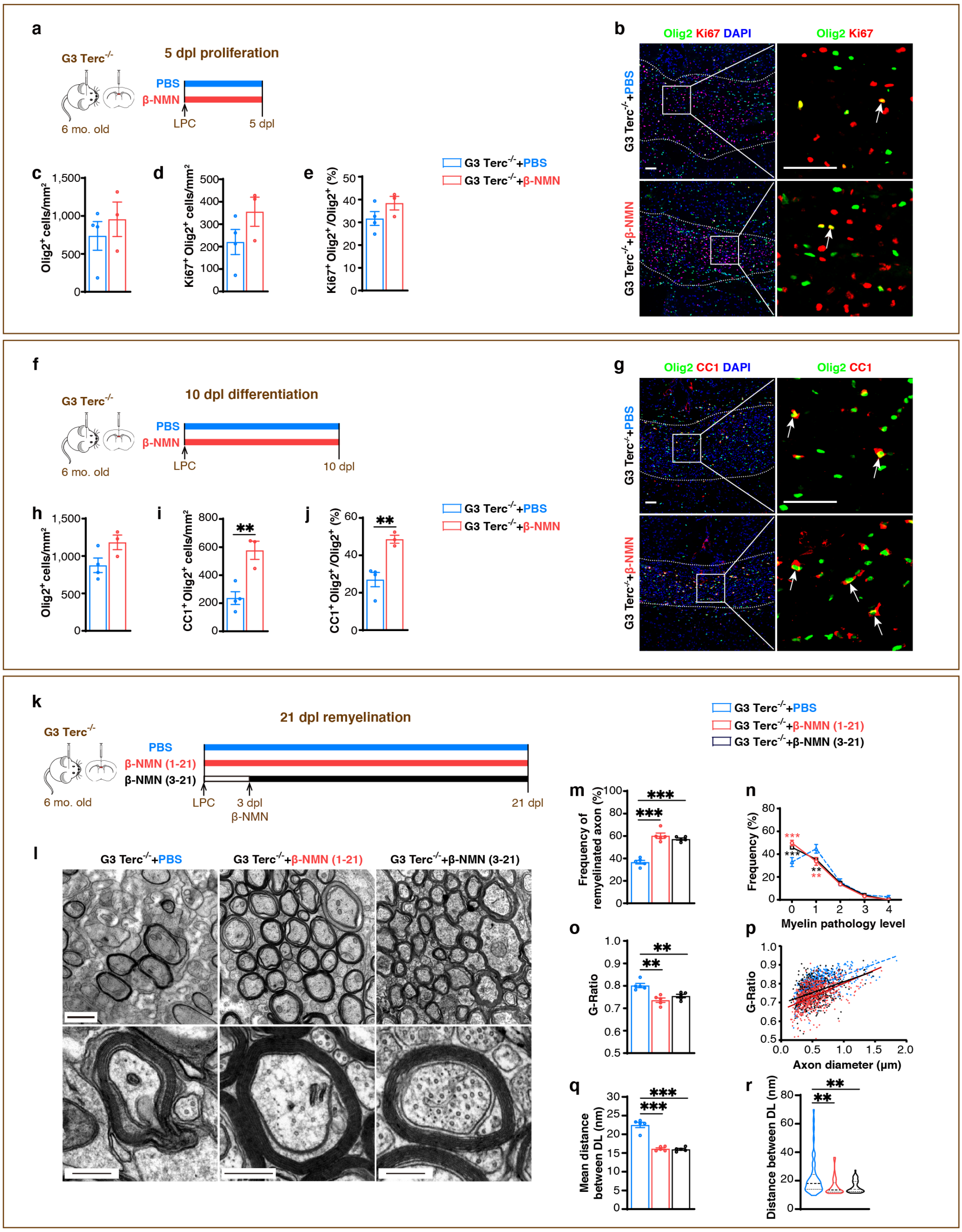

Supplementary Fig.12 | Immediate and delayed *in vivo* NAD+ supplementation enhances remyelination in the aged mice. a, Experiment design for testing the impact of β-NMN on OPC proliferation *in vivo*. **b-e**, Images and quantification of the densities of oligodendrocyte lineage cells (**c**) and proliferating OPCs (**d**) and the proportion of proliferating OPCs among all oligodendrocyte lineage cells (**e**) within the demyelination lesions at 5 dpl (n=4 for the G3 Terc-/-+PBS group, n=3 for the G3 Terc-/-+β-NMN group). Scale bar, 50 μm. **f**, Experiment design for testing the impact of β-NMN on OPC differentiation *in vivo*. **g-j**, Images and quantification of

the densities of oligodendrocyte lineage cells (**h**) and differentiated oligodendrocytes (**i**) and the proportion of differentiated oligodendrocytes among all oligodendrocyte lineage cells (**j**) within the demyelination lesions at 10 dpl (n=4 for the G3 Terc$^{-/-}$+PBS group, n=3 for the G3 Terc$^{-/-}$+β-NMN group). Scale bar, 50 μm. **k**, Experiment design for testing the impact of β-NMN on ultrastructural remyelination efficiency *in vivo* at 21 dpl. Note β-NMN administration was carried out at 2 time points. **l**, TEM micrographs within the lesions at 21 dpl. Scale bar, 1 μm for the upper panel and 200 nm for the lower panel. **m**-**r**, Quantification of the percentage of remyelinated axons (**m**), myelin pathology level (**n**), average G-Ratio (**o**), individual G-Ratio distribution (**p**, linear regression) and distance between DL (**q** and **r**) within the lesions at 21 dpl (n=5). All data are presented as mean ± SEM. The center, upper and lower line represent the median, upper and lower quartiles, respectively (**r**). *p<0.05, **p<0.01, ***p<0.001 by two-tailed t test (**c**-**e**, **h**-**j**), one-way ANOVA followed by Tukey's post hoc test (**m**, **o**, **q**, **r**) or two-way repeated ANOVA followed by Sidak's post hoc test (**n**). In all instances ***p<0.001. *n.s.* no significance. In (**i**), **p=0.0068; in (**j**), **p=0.0067; in (**n**), **p=0.002 (grade 1, PBS vs. β-NMN (1-21)), **p=0.009 (grade 1, PBS vs. β-NMN (3-21)); in (**o**), **p=0.001 (PBS vs. β-NMN (1-21)), **p=0.005 (PBS vs. β-NMN (3-21)); in (**r**), **p=0.003 (PBS vs. β-NMN (1-21)), **p=0.001 (PBS vs. β-NMN (3-21)).

Supplementary Fig.13

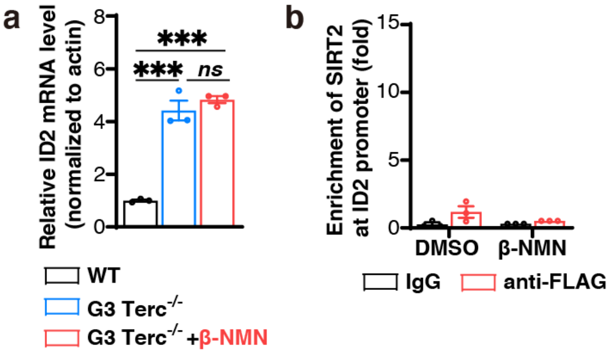

Supplementary Fig.13 | NAD+ supplementation neither regulates mRNA of ID2 nor enriches SIRT2 at ID2 promoter in OPCs *in vitro*. a, Relative mRNA level of ID2 in primary cultured OPCs with/without β-NMN treatment and OPCs were from WT or G3 Terc$^{-/-}$ mice (n=3). **b**, ChIP-qPCR assessment of the enrichment of SIRT2 at the promoter region of ID2 in OLN93 cell line overexpressing SIRT2 (n=3). All data are presented as mean ± SEM. *p<0.05, **p<0.01, ***p<0.001 by one-way ANOVA followed by Tukey's post hoc test (**a**) or two-way repeated ANOVA followed by Sidak's post hoc test (**b**). In all instances ***p<0.001. *n.s.* no significance.

Supplementary Fig.14

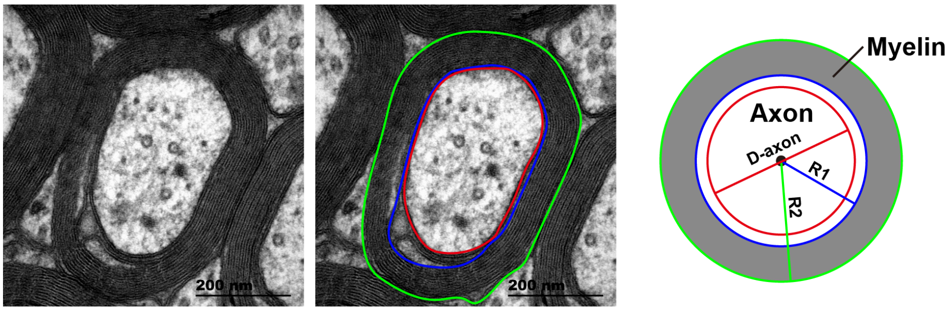

Supplementary Fig.14 | The schematic diagram of G-Ratio measurement. R1: radius of inner circle (blue); area1: area of inner circle (blue); R2: radius of outer circle (green); area2: area of outer circle (green); D-axon: diameter of axon (red); area3: area of axon (red).

$\mathrm{R1} = \sqrt{\mathrm{area1}/\pi};\ \mathrm{R2} = \sqrt{\mathrm{area2}/\pi};\ \mathrm{G-Ratio} = \mathrm{R1}/\mathrm{R2};\ \mathrm{D-axon} = 2\sqrt{\mathrm{area3}/\pi}$
