# Supplementary material for: Restoring nuclear entry of Sirtuin 2 in oligodendrocyte progenitor cells promotes remyelination during ageing
Source: Nat Commun. 2022 Mar 9;13:1225. doi: 10.1038/s41467-022-28844-1 (PMC8907257; doi:10.1038/s41467-022-28844-1)
Supplement: Supplementary file 4 — Description of Additional Supplementary Files. [file 41467_2022_28844_MOESM4_ESM.pdf]

Description of Additional Supplementary Files

File Name: Supplementary Data 1
Description: **Graded Myelin Age-related Ultrastructural Changes.**

File Name: Supplementary Data 2
Description: **Metabolome comparison of WT and G3 Terc$^{-/-}$ OPCs.**

File Name: Supplementary Data 3
Description: P**roteome comparison of rat OPCs treatment with β-NMN or DMSO.**

File Name: Supplementary Data 4
Description: **Sequence of primers.**
